# Chinese Medicinal Herbs for Childhood Pneumonia: A Systematic Review of Effectiveness and Safety

**DOI:** 10.1155/2013/203845

**Published:** 2013-03-10

**Authors:** Qianchun Yang, Darong Wu, Wei Mao, Xusheng Liu, Kun Bao, Qizhan Lin, Fuhua Lu, Chuan Zou, Chuang Li

**Affiliations:** ^1^The Second Clinical Medical College, Guangzhou University of Chinese Medicine, Guangzhou 510405, China; ^2^Department of Nephropathy Medicine, Guangdong Provincial Hospital of Chinese Medicine, Guangzhou 510120, China; ^3^Outcome Assessment Branch, Department of Clinical Epidemiology, Guangdong Provincial Hospital of Chinese Medicine, Guangzhou 510120, China; ^4^Department of Nephrology Laboratory, Guangdong Provincial Hospital of Chinese Medicine, Guangzhou 510120, China

## Abstract

*Objective*. To assess the efficacy and safety of Chinese medicinal herbs for Childhood Pneumonia. *Methods*. We included randomized controlled trials (RCTs). The searched electronic databases included PubMed, the Cochrane Central Register of Controlled Trials, EMBASE, CBM, CNKI, and VIP. All studies included were assessed for quality and risk bias. Review Manager 5.1.6 software was used for data analyses, and the GRADEprofiler software was applied to classify the systematic review results. *Results*. Fourteen studies were identified (*n* = 1.824). Chinese herbs may increase total effective rate (risk ratio (RR) 1.18; 95% confidence interval (CI), 1.11–1.26) and improve cough (total mean difference (MD), −2.18; 95% CI, (−2.66)–(−1.71)), fever (total MD, −1.85; 95% CI, (−2.29)–(−1.40)), rales (total MD, −1.53; 95% CI, (−1.84)–(−1.23)), and chest films (total MD, −3.10; 95% CI, (−4.11)–(−2.08)) in Childhood Pneumonia. Chinese herbs may shorten the length of hospital stay (total MD, −3.00; 95% CI, (−3.52)–(−2.48)), but no significant difference for adverse effects (RR, 0.39; 95% CI, 0.09–1.72) was identified. *Conclusion*. Chinese herbs may increase total effective rate and improve symptoms and signs. However, large, properly randomized, placebo-controlled, double-blind studies are required.

## 1. Introduction

Childhood Pneumonia is an acute virus, bacterial, or fungal respiratory infection that affects the lungs [1]. The symptoms and signs of Childhood Pneumonia include cough, fever, rapid or difficult breathing, loss of appetite, and lower chest wall indrawing [2]. Auscultation reveals rales. However, severe Childhood Pneumonia is defined as cough or difficult breathing combined with lower chest wall indrawing [3]. Childhood Pneumonia has been identified as a mixed viral-bacterial infection in 23%–33% of cases [4]. Respiratory syncytial virus is the most common viral cause of children pneumonia, and *Streptococcus pneumoniae* is the most common cause of bacterial pneumonia in children [5]. Each year, pneumonia kills an estimated 1.4 million children <5 years of age, accounting for 18% of all deaths worldwide [6]. Pneumonia is the most common reason for hospitalization in children <2 years of age [7]. The cost of antibiotic treatment for all children with pneumonia in 42 of the poorest countries is estimated to be about US $600 million per year. The estimated incidence rates are 0.29 and 0.05 episodes per child-year in low-income and high-income countries, respectively. Approximately 156 million new episodes occur each year, the majority in India (43 million), China (21 million), Pakistan (10 million), Bangladesh, Indonesia, and Nigeria (6 million each) [8].

Administering appropriate antibiotics at the early stage of pneumonia improves outcomes, particularly when the causative agent is bacterial [9]. However, antibiotic treatment of pneumonia in children remains mostly empirical because determining the etiologic pathogen is difficult in this age group [10]. According to the results of a questionnaire, the following agents have been used against Childhood Pneumonia: ampicillin, ampicillin/sulbactam, second/third generation cephalosporins, azithromycin, vancomycin, clindamycin, and linezolid. In subsequent analyses, we categorized ampicillin, ampicillin/sulbactam, and cephalosporins as beta-lactam antibiotics and vancomycin, clindamycin, and linezolid as antimethicillin-resistant* Staphylococcus aureus* antibiotics. Respondents were asked to select the duration of antibiotic therapy they would recommend for uncomplicated and parapneumonic empyema cases using the following categories: 3–5 days, 6-7 days, 8–10 days, 11–14 days, 15–21 days, and >21 days [11]. Empirical antibiotic administration is relied upon in most instances to meet the public health goal of reducing child mortality due to pneumonia. This is necessary in view of the inability of most commonly available laboratory tests to identify causative pathogens. Empirical antibiotic administration is the main treatment for Childhood Pneumonia. Multiple antibiotics are prescribed for treating pneumonia, so it is important to know which work best for pneumonia in children [12].

Traditional Chinese Medicine (TCM) follows a particular theoretical and methodological approach to estimate the cause of a disease, leading to diagnosis and treatment [13]. Pneumonia is equivalent to the TCM cough category. Ma Xing Shi Gan Tang, San Ao Tang, Zhi Sou San, and other self-developed TCM prescriptions are Chinese medicinal formulas that have been used to treat Childhood Pneumonia for many years. In recent years, preparations of Chinese herbal medicines, such as Tanreqing injection, Chuanhuning injection, and Reduning injection have been used to treat Childhood Pneumonia in China. Chinese herbal medicine formulas function to clear heat, resolve phlegm, ventilate the lungs, dissipate phlegm, relieve cough, and reduce sputum. The function of Chinese herbal medicine preparations is to clear heat and remove toxicity. A study showed that the pharmacological action of Ma Xing Shi Gan Tang includes antiasthmatic, antitussive, and antiviral effects as well as bacteriostatic and immunoregulatory functions [14].

Although these formulae and other Chinese herbal medicine preparations have been used widely to treat Childhood Pneumonia in China, their effects and safety have not been reviewed systematically.

## 2. Methods

### 2.1. Criteria for Considering Studies for this Paper

#### 2.1.1. Types of Studies

Only randomized controlled trials (RCTs) were included. 

#### 2.1.2. Types of Participants

Studies that enrolled patients with pneumonia in children who had cough, fever >37.5°C, raised respiratory rate, lower chest wall indrawing, rales, and changes on chest films were included. Patients with Childhood Pneumonia of either gender, any ethnic group, and ages of 1 month to 18 years were included. Studies were excluded if they included children suffering from other debilitating diseases. 

#### 2.1.3. Types of Interventions

There were Chinese medicinal herbs versus other drugs, formulas, and placebo alone; Chinese medicinal herbs plus basic therapy versus basic therapy. Antibiotics were one of the main basic therapies for Childhood Pneumonia. Prohibited or suspended Chinese herbal preparations were excluded.

#### 2.1.4. Types of Outcome Measures

Primary outcomes included mortality and total effective rate (e.g., ratio of signs and symptoms improvement or recovery); secondary outcomes included time to clinical recovery (e.g., cough, fever, rales, and chest films), relapse rate, length of hospital stay, and adverse effects (e.g., nausea, diarrhea, vomiting, and gastrointestinal bleeding). TCM outcomes such as tongue coat, pulse condition, and economic index were included. 

### 2.2. Search Methods for Identification Studies

We searched for all relevant studies in the following electronic databases: PubMed (1966–July 2012), the Cochrane Central Register of Controlled Trials, EMBASE (1980–July 2012), the Chinese Biomedicine Database (CBM) (1976–July 2012), Chinese National Knowledge Internet (CNKI) (1979–July 2012), and Chinese Biomedical Journals (VIP) (1989–July 2012). All studies included were analyzed according to Cochrane Handbook criteria. The following search terms were used: (Chinese herbs OR Chinese traditional herbs OR Chinese medicinal herbs OR traditional Chinese herbs OR Chinese herbal medicines) AND (child pneumonia OR children pneumonia OR Childhood Pneumonia OR Pediatric Pneumonia OR Infantile Pneumonia). We conducted a manual search for the Journal of Guangzhou University of Traditional Chinese Medicine. We attempted to contact original authors to obtain the protocol for the studies.

### 2.3. Data Collection and Analysis

#### 2.3.1. Study Selection

Two review authors independently browsed the titles and abstracts of all articles identified by the literature search. The same review authors independently estimated whether the trials met the inclusion criteria. Disagreements were resolved by discussion or consultation with a third author. We assessed abstracts from the initial search independently to identify studies that met the inclusion criteria. We telephone-interviewed authors of Chinese language articles and emailed the original authors of English articles to identify the randomization procedure and other methodological questions to ensure that the included studies were RCTs. If the required information was not available or if the required information did not meet the inclusion criteria, the article was excluded.

#### 2.3.2. Data Extraction and Management

We extracted data including methodological details and data from publications using a data extraction form. We extracted data on study characteristics, including methods, participants, interventions, and outcomes. There were no disagreements among the authors.

We extracted the formulation contents of the included studies, and the names of the herbs are provided in three languages (e.g., Chinese, Latin, and English) in Table 1.

#### 2.3.3. Assessment of Risk of Bias in Included Studies

The following items were independently assessed by our authors using the risk of bias assessment tool. (1) Was there adequate sequence generation (selection bias)? (2) Was allocation adequately concealed (selection bias)? (3) Was knowledge of the allocated interventions adequately prevented during the study (e.g., participants and personnel, outcome assessors) (detection bias)? (4) Were incomplete outcome data adequately addressed (attrition bias)? (5) Are reports of the study free of suggesting selective outcome reporting (reporting bias)? (6) Was the study apparently free of other problems that could put it at risk for bias?

#### 2.3.4. Measures of Treatment Effect

Data analyses were performed using the Cochrane Collaboration's RevMan software, version 5.1.6. Results are expressed as risk ratios (RR) and 95% confidence intervals (CIs) for dichotomous outcomes (e.g., mortality, effective rate, adverse effects, and relapse rate) and as mean differences (MD) with 95% CIs for continuous outcomes (such as time to clinical recovery and length of hospital stay).

#### 2.3.5. Assessment of Heterogeneity

Heterogeneity was analyzed using the chi-square test on *n* − 1 degrees of freedom, and an alpha of 0.05 was used for statistical significance with the *I* ^2^ test. *I* ^2^ values of 25, 50, and 75% corresponded to low, medium, and high levels of heterogeneity, respectively.

#### 2.3.6. Data Synthesis

We used fixed-effects and random-effects models for the pooled data analysis. We performed a pooled analysis for the 14 studies.

#### 2.3.7. Subgroup Analyses

Subgroup analyses were performed according to formula type and Chinese medicinal herb preparation type, using the same comparators (e.g., same types of antibiotics).

#### 2.3.8. Sensitivity Analyses

Sensitivity analyses were conducted by excluding low-quality studies (based on descriptions of randomization, allocation concealment, blinded assessment of outcomes, and description/analyses of withdrawals and dropouts) and a comparison of the merger analysis results for the fixed- and random-effects models.

## 3. Results

### 3.1. Search Results

**Figure 1:**
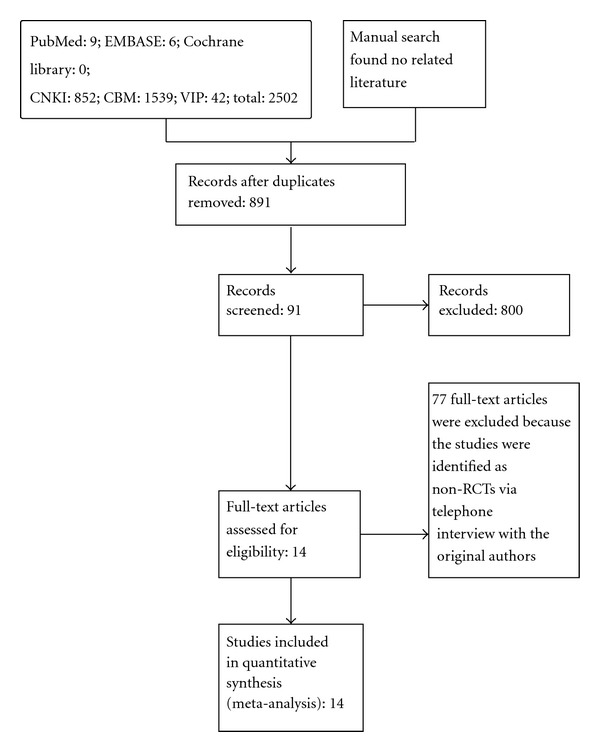
Summary of the search results in a flow diagram.

An initial search identified 2,502 potentially relevant articles. Of these, 15 were in the English database. A total of 891 articles were initially included after duplicate publications were removed and any obviously irrelevant were excluded; 800 articles were later excluded, because they did not meet the inclusion criteria. Of the 91 potentially eligible reports, 77 were excluded for further assessment because telephone interviews with the original authors revealed that they were not RCTs. Therefore, 14 studies (1,824 participants) were included in this paper. All 14 studies were published in Chinese (Figure 1). 

### 3.2. Included Studies

#### 3.2.1. Participants

The ratio of male to female participants in the 14 studies was 879/615 [15–21, 23–28]. One study [22] did not report the number of males and females. In total, 1,824 children were included in the 14 studies, and all were from China [15–28]. The ages of the patients were 1 month–15 years. The average size of the trials was 130 participants (range 60–200 participants).

#### 3.2.2. Inclusion Criteria

The diagnostic criteria for childhood pneumonia in all studies included fever >37.5°C, chest recession, increased respiratory rate, cough, rales, or difficulty breathing combined with fast breathing and a change on chest films. 

#### 3.2.3. Intervention

Chinese medicinal herbs interventions were given as oral decoctions or intravenous infusions. The longest therapy duration was 3 weeks, and the shortest was 5 days. Follow-up duration was not mentioned by any of the authors. All 14 studies compared Chinese medicinal herbs plus basic therapy versus basic therapies. 

#### 3.2.4. Outcomes

All 14 studies [15–28] reported the total effective rate; seven studies [15, 17, 18, 21, 23, 25, 28] reported clinical recovery of cough, fever, rales, and chest films; four [16, 22, 24, 26] reported clinical recovery of cough, fever, and rales; one [27] reported clinical recovery of fever; three [15, 21, 22] reported adverse effects (e.g., nausea, vomiting, diarrhea, and gastrointestinal bleeding); and one [28] reported the length of hospital stay. The description of studies is detailed in the characteristics of included studies in Table 2.

### 3.3. Risk of Bias in Included Studies

**Figure 2:**
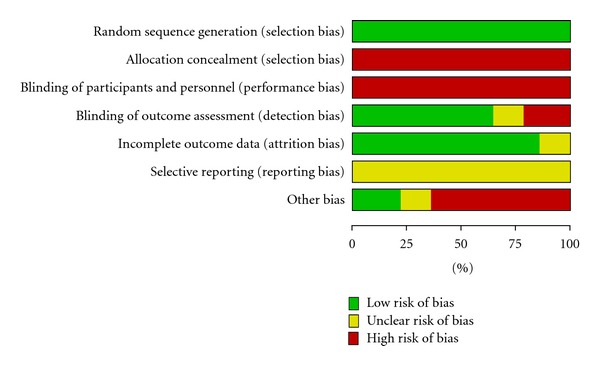
Methodological quality. Judgments about each item are presented as percentages across all included studies.

**Figure 3:**
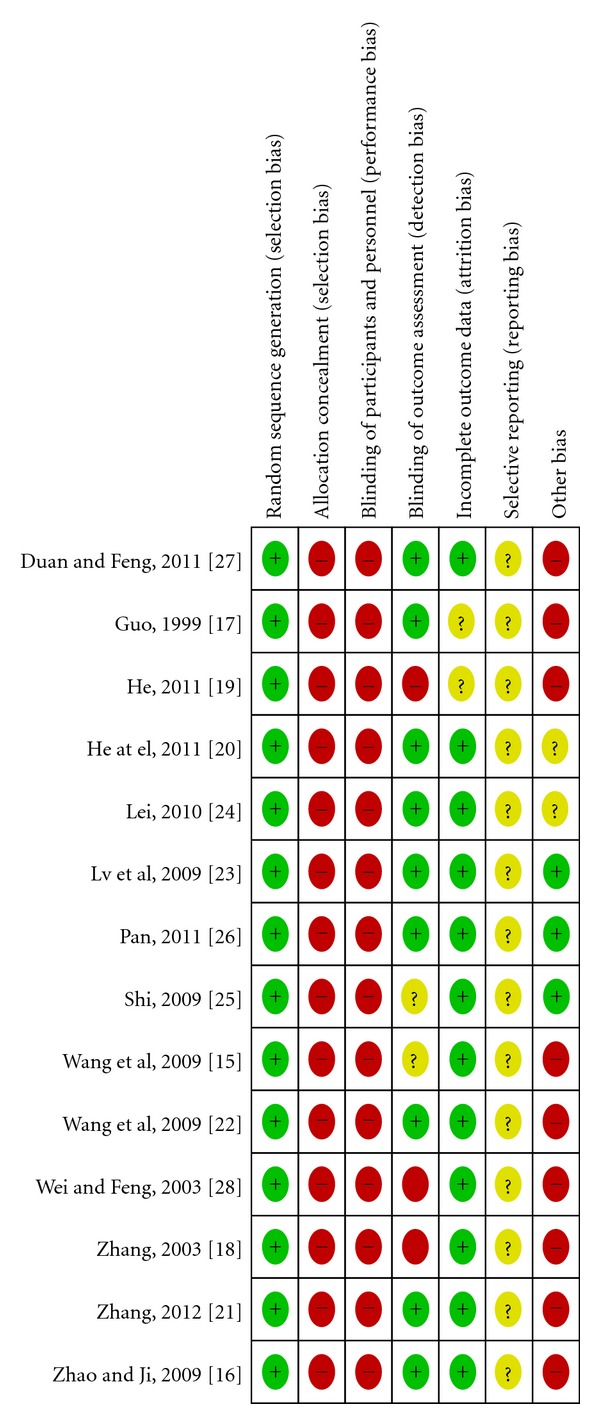
Methodological quality summary.

The methodological quality of each study's randomization sequence, allocation concealment, blinding, incomplete outcome data, selective outcome reporting, and potential threats are summarized in Figures 2 and 3.

#### 3.3.1. Randomization and Allocation Concealment

Ten studies [15–20, 23, 25–27] reported using a random-number table, and four [21, 22, 24, 28] reported using a computer-generated random-number table. None of the trials used allocation concealment. Therefore, all studies had a high risk of selection bias.

#### 3.3.2. Blinding

Ten studies [15–17, 20–24, 26, 27] used single blinding (outcome assessment was blinded). We interviewed the original authors by telephone to determine blinding because the blinding methods were not described. Thus, these studies had a low risk of performance bias and low detection bias. The other studies [18, 19, 25, 28] did not use blinding methods and had a high risk of performance bias or a strong detection bias.

#### 3.3.3. Flow of Participants and Intention-to-Treat

None of the studies reported withdrawal, dropout, and/or loss during followup. The method of handling missing data regarding intention-to-treat or per-protocol analysis was not addressed.

#### 3.3.4. Selective Reporting (Reporting Bias)

No detailed evidence of selective reporting was found in any of the 14 studies [15–28]. However, we believed there is a high risk of selective reporting bias because we were unable to compare the protocol with published studies.

#### 3.3.5. Other Potential Sources of Bias

None of the 14 studies [15–28] did not describe patient compliance. The appropriateness of the statistical analyses used was assessed, and the methods of all studies were considered appropriate. Although we conducted comprehensive searches and tried to avoid bias, we could not exclude potential publication bias because all 14 studies were published in China.

## 4. Effects of Interventions

All studies compared Chinese medicinal herbs plus basic therapy to basic therapy alone. The Chinese medicinal herb treatments included the modified Ma Xing Shi Gan Tang formula, the San Ao Tang formula, the Zhi Sou San formula, a self-developed TCM prescription, Tanreqing injection, Reduning injection, and Chuanhuning injection. Antibiotics were one of main basic therapies for Childhood Pneumonia.

### 4.1. Total Effective Rate

**Figure 4:**
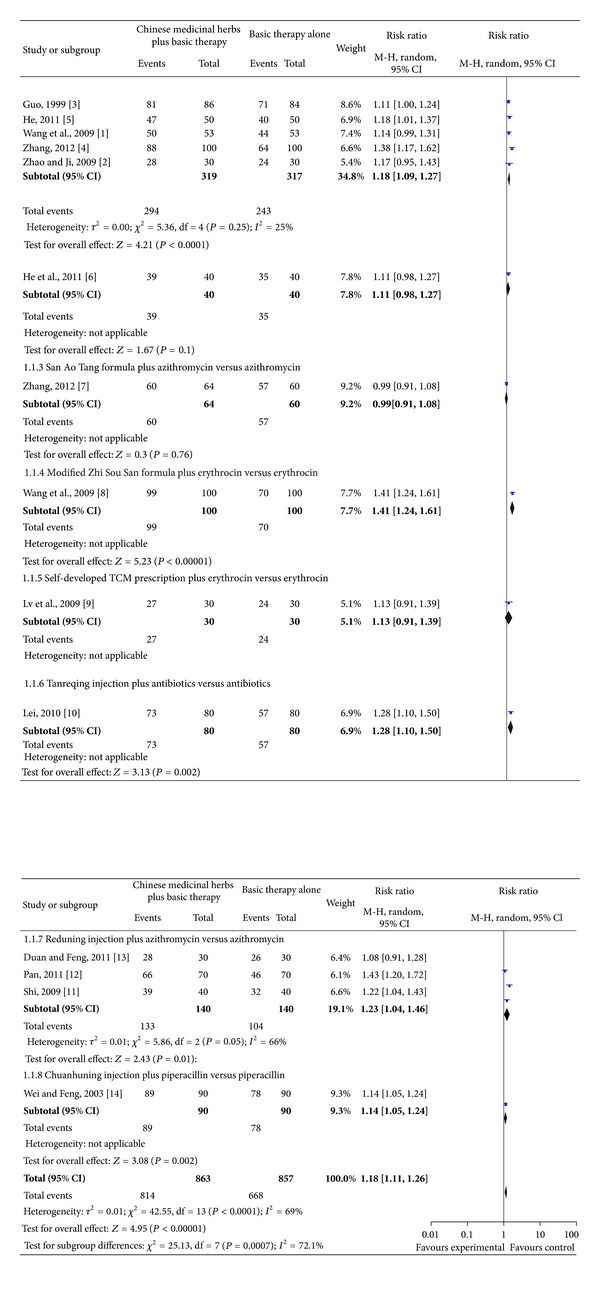
Comparison. Chinese medicinal herbs plus basic therapy versus basic therapy: outcome 1 total effective rate.

A significant increase in total effective rate was observed with Chinese medicinal herbs plus basic therapy versus basic therapy (Figure 4, analysis 1.1; RR, 1.18; 95% CI, 1.11–1.26). 

Subgroup five studies [15–19] compared the modified Ma Xing Shi Gan Tang formula plus azithromycin versus azithromycin and showed a significant increase in total effective rate (Figure 4; analysis 1.1.1 of analysis 1.1; RR, 1.18; 95% CI, 1.09–1.27). 

Subgroup one study [20] compared the modified Ma Xing Shi Gan Tang formula plus ceftazidime versus ceftazidime and showed no difference in total effective rate (Figure 4; analysis 1.1.2 of analysis 1.1; RR, 1.11; 95% CI, 0.98–1.27). 

Subgroup one study [21] compared the San Ao Tang formula plus azithromycin versus azithromycin and showed no difference in total effective rate (Figure 4; analysis 1.1.3 of analysis 1.1; RR, 0.99; 95% CI, 0.91–1.08). 

Subgroup one study [22] compared the modified Zhi Sou San formula plus erythrocin versus erythrocin and showed a significant increase in total effective rate (Figure 4; analysis 1.1.4 of analysis 1.1; RR, 1.41; 95% CI, 1.24–1.61). 

Subgroup one study [23] compared a self-developed TCM prescription plus erythrocin versus erythrocin and showed no difference in total effective rate (Figure 4; analysis 1.1.5 of analysis 1.1; RR, 1.13; 95% CI, 0.91–1.39).

Subgroup one study [24] compared Tanreqing injection plus antibiotics versus antibiotics and showed a significant difference in total effective rate (Figure 4; analysis 1.1.6 of analysis 1.1; RR, 1.28; 95% CI, 1.10–1.50).

Subgroup three studies [25–27] compared Reduning injection plus azithromycin versus azithromycin and showed a significant difference in total effective rate (Figure 4; analysis 1.1.7 of analysis 1.1; RR, 1.23; 95% CI, 1.04–1.46). 

Subgroup one study [28] compared Chuanhuning injection plus piperacillin versus piperacillin and showed a significant difference in total effective rate (Figure 4; analysis 1.1.8 of analysis 1.1; RR, 1.14; 95% CI, 1.05–1.24).

### 4.2. Adverse Effects

**Figure 5:**
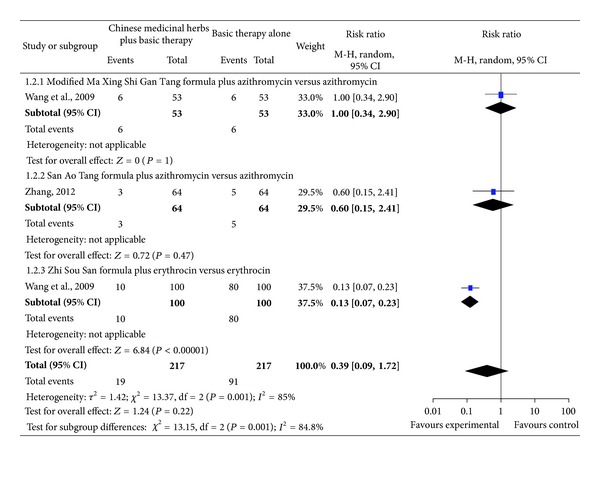
Comparison. Chinese medicinal herbs plus basic therapy versus basic therapy: outcome 2 adverse effects.

Three studies [15, 21, 22] compared Chinese medicinal herbs plus basic therapy versus basic therapy and showed no difference in adverse effects (Figure 5; analysis 1.2; RR, 0.39; 95% Cl, 0.09–1.72). 

Subgroup one study [15] compared the modified Ma Xing Shi Gan Tang formula plus azithromycin versus azithromycin and showed no difference in adverse effects (Figure 5; analysis 1.2.1 of analysis 1.2; RR, 1.00; 95% CI, 0.34–2.90).

Subgroup one study [21] compared the San Ao Tang formula plus azithromycin versus azithromycin and showed no difference in adverse effects (Figure 5; analysis 1.2.2 of analysis 1.2; RR, 0.60; 95% CI, 0.15–2.41).

Subgroup one study [22] compared the modified Zhi Sou San formula plus erythrocin versus erythrocin and showed a significant decrease in adverse effects (Figure 5; analysis 1.2.3 of analysis 1.2; RR, 0.13; 95% CI, 0.07–0.23).

### 4.3. Time (Day) to Clinical Recovery Including Cough, Fever, Rales, and Chest Films

**Figure 6:**
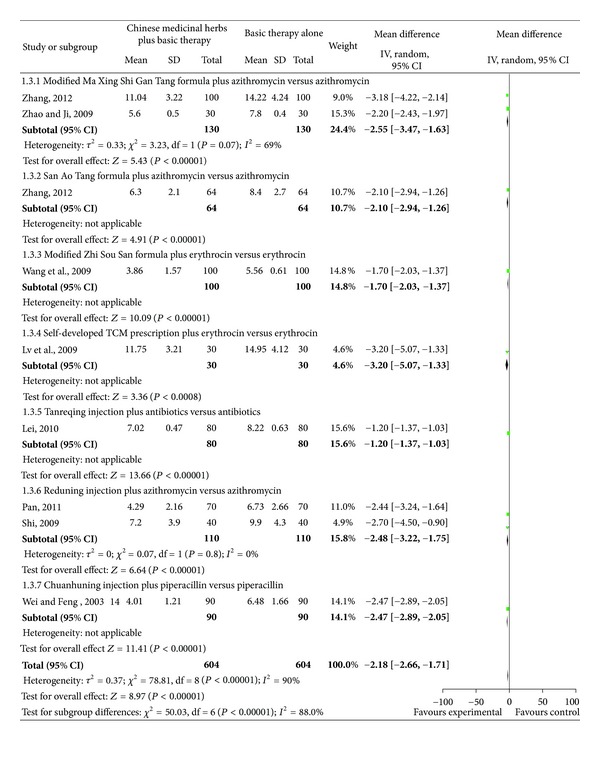
Comparison. Chinese Medicinal herbs plus basic therapy versus basic therapy: outcome 3 cough.

**Figure 7:**
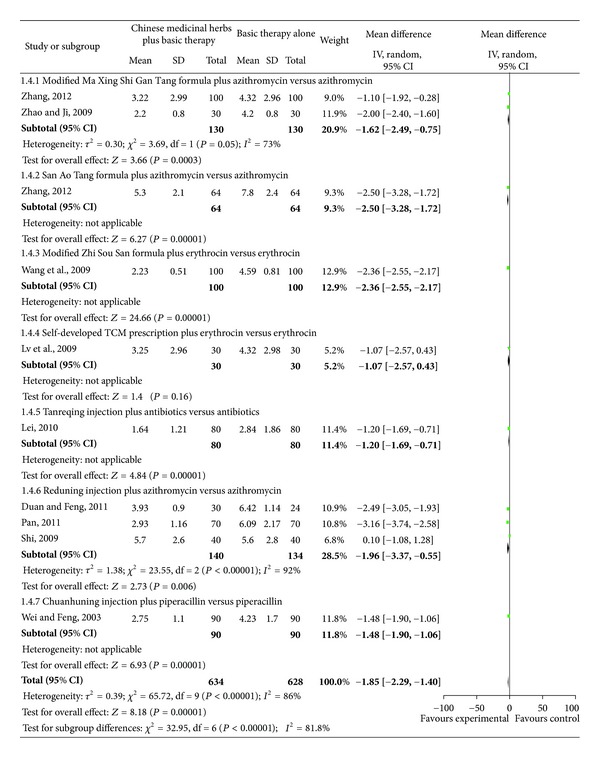
Comparison. Chinese medicinal herbs plus basic therapy versus basic therapy: outcome 4 fever.

**Figure 8:**
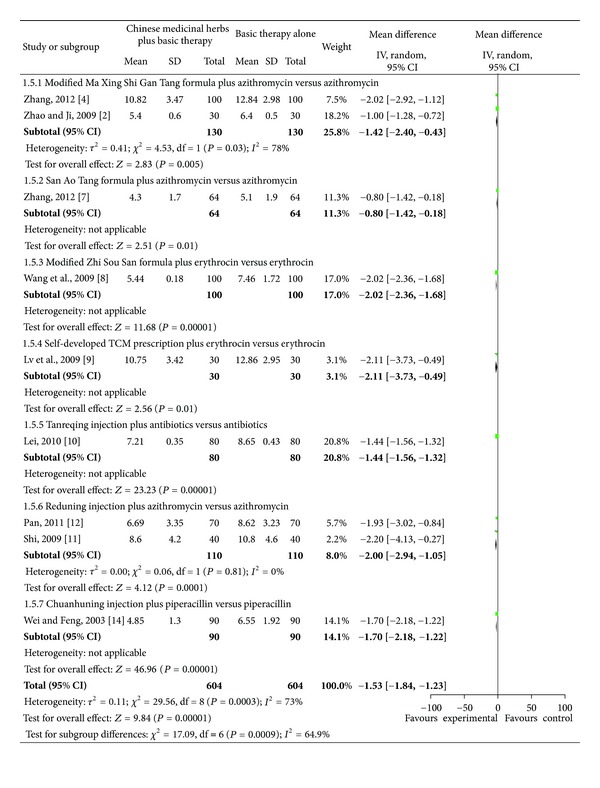
Comparison. Chinese medicinal herbs plus basic therapy versus basic therapy: outcome 5 rales.

**Figure 9:**
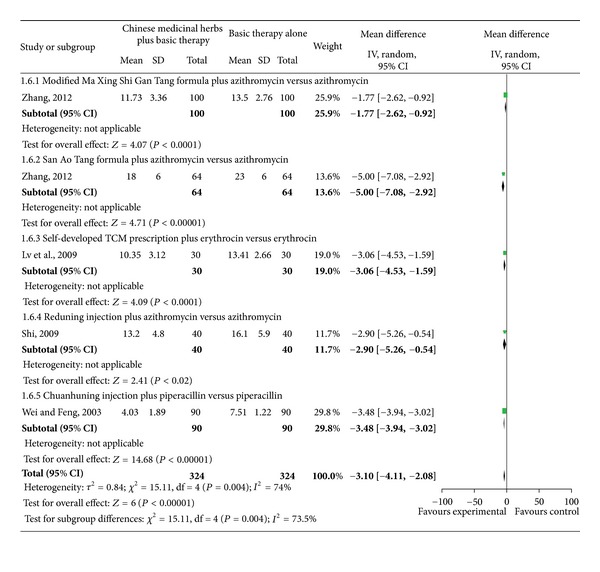
Comparison. Chinese medicinal herbs plus basic therapy versus basic therapy: outcome 6 chest films.

Studies investigating Chinese medicinal herbs plus basic therapy versus basic therapy showed a significant difference in cough (Figure 6; analysis 1.3; total MD, −2.18; 95% Cl, (−2.66)–(−1.71)), fever (Figure 7; analysis 1.4; total MD, −1.85; 95% Cl, (−2.29)–(−1.40)), rales (Figure 8; analysis 1.5; total MD, −1.53; 95% Cl, (−1.84)–(−1.23)), and chest films (Figure 9; analysis 1.6; total MD, −3.10; 95% Cl, (−4.11)–(−2.08)). 

Subgroup two studies [16, 18] compared the modified Ma Xing Shi Gan Tang formula plus azithromycin versus azithromycin and showed a significant difference for cough (Figure 6; analysis 1.3.1 of analysis 1.3; MD, −2.55; 95% Cl, (−3.47)–(−1.63)), fever (Figure 7; analysis 1.4.1 of analysis 1.4; MD, −1.62; 95% Cl, (−2.49)–(−0.75)), and rales (Figure 8; analysis 1.5.1 of analysis 1.5; MD, −1.42; 95% Cl, (−2.40)–(−0.43)). Subgroup one study [18] compared the modified Ma Xing Shi Gan Tang formula plus azithromycin versus azithromycin and showed a significant difference on chest films (Figure 9; analysis 1.6.1 of analysis 1.6; MD, −1.77; 95% Cl, (−2.62)–(−0.92)).

Subgroup one study [21] compared the San Ao Tang formula plus azithromycin versus azithromycin and showed a significant difference in cough (Figure 6; analysis 1.3.2 of analysis 1.3; MD, −2.10; 95% Cl, (−2.94)–(−1.26)), fever (Figure 7; analysis 1.4.2 of analysis 1.4; MD, −2.50; 95% Cl, (−3.28)–(−1.72)), rales (Figure 8; analysis 1.5.2 of analysis 1.5; MD, −0.80; 95% Cl, (−1.42)–(−0.18)), and chest films (Figure 9; analysis 1.6.2 of analysis 1.6; MD, −5.00; 95% Cl, (−7.08)–(−2.92)).

Subgroup one study [22] compared the modified Zhi Sou San formula plus erythrocin versus erythrocin and showed a significant difference in cough (Figure 6; analysis 1.3.3 of analysis 1.3; MD, −1.70; 95% CI, (−2.03)–(−1.37)), fever (Figure 7 analysis 1.4.3 of analysis 1.4; MD, −2.36; 95% Cl, (−2.55)–(−2.17)), and rales (Figure 8 analysis 1.5.3 of analysis 1.5; MD, −2.02; 95% Cl, (−2.36)–(−1.68)).

Subgroup one study [23] compared a self-developed TCM prescription plus erythrocin versus erythrocin and showed a significant difference in cough (Figure 6 analysis 1.3.4 of analysis 1.3; MD, 3.20; 95% CI, (−5.07)–(−1.33)), rales (Figure 8 analysis 1.5.4 of analysis 1.5; MD, −2.11; 95% Cl, (−3.73)–(−0.49)), and chest films (Figure 9 analysis 1.6.3 of analysis 1.6; MD, −3.06; 95% Cl, (−4.53)–(−1.59)), but no difference in fever (Figure 7 analysis 1.4.4 of analysis 1.4; MD, −1.07; 95% Cl, −2.57–0.43).

Subgroup one study [24] compared Tanreqing injection plus antibiotics versus antibiotics and showed a significant difference in cough (Figure 6 analysis 1.3.5 of analysis 1.3; MD, −1.20; 95% CI, (−1.37)–(−1.03)), fever (Figure 7 analysis 1.4.5 of analysis 1.4; MD, −1.20; 95% Cl, (−1.69)–(−0.71)), and rales (Figure 8 analysis 1.5.5 of analysis 1.5; MD, −1.44; 95% Cl, (−1.56)–(−1.32)).

Subgroup two studies [25, 26] compared Reduning injection plus azithromycin versus azithromycin and showed a significant difference in cough (Figure 6 analysis 1.3.6 of analysis 1.3; MD, −2.48; 95% CI, (−3.22)–(−1.75)) and rales (Figure 8 analysis 1.5.6 of analysis 1.5; MD, −2.00; 95% CI, (−2.94)–(−1.05)). Subgroup three studies [25–27] compared Reduning injection plus azithromycin versus azithromycin and showed a significant difference in fever (Figure 7 analysis 1.4.6 of analysis 1.4; MD, −1.96; 95% CI, (−3.37)–(−0.55)). Subgroup one study [25] compared Reduning injection plus azithromycin versus azithromycin and showed a significant difference on chest films (Figure 9 analysis 1.6.4 of analysis 1.6; MD, −2.90; 95% CI, (−5.26)–(−0.54)).

Subgroup one study [28] compared Chuanhuning injection plus piperacillin versus piperacillin and showed a significant difference in cough (Figure 6 analysis 1.3.7 of analysis 1.3; MD, −2.47; 95% CI, (−2.89)–(−2.05)), fever (Figure 7 analysis 1.4.7 of analysis 1.4; MD, −1.48; 95% CI, (−1.90)–(−1.06)), rales (Figure 8 analysis 1.5.7 of analysis 1.5; MD, −1.70; 95% CI, (−2.18)–(−1.22)), and chest films (Figure 9 analysis 1.6.5 of analysis 1.6; MD, −3.48; 95% CI, (−3.94)–(−3.02)).

### 4.4. Length of Hospital Stay

**Figure 10:**
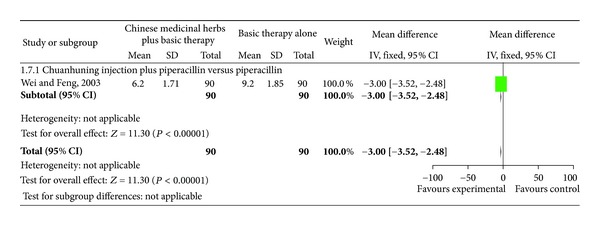
Comparison. Chinese medicinal herbs plus basic therapy versus basic therapy: outcome 7 length of hospital stay.

One study [28] compared Chuanhuning injection plus piperacillin versus piperacillin and showed a significant difference in length of hospital stay (Figure 10 analysis 1.7; total MD, −3.00; 95% CI, (−3.52)–(−2.48)).

### 4.5. Other Outcomes

Mortality, relapse rate, TCM outcomes (e.g., tongue coat and pulse condition), and economic index were not reported in any of the 14 studies.

## 5. GRADE Quality of Evidence

The “GRADEprofiler” of the Cochrane Collaboration Network was used to classify the systematic review results. The quality of evidence was low to very low (Table 3).

## 6. Discussion

Based on the 14 [15–28] RCTs conducted in China, Chinese medicinal herbs increased total effective rates (e.g., ratio of signs and symptoms improvement or recovery) and improved clinical symptoms and signs (e.g., cough, fever, rales, and chest films). However, the evidence that Chinese medicinal herbs decreased adverse effects, mortality, or improved TCM outcomes (e.g., tongue coat and pulse condition) was insufficient. The quality of the evidence was weak due to selective bias, measurement bias, selective reporting bias, and imprecision. Therefore, evidence from the included studies was not enough to make any recommendations.

First, randomization was mentioned in all 14 studies. However, one study [25] described the randomization method in detail, whereas 13 did not [15–24, 26–28]. We interviewed the authors by telephone and determined that a random number table or a computer-generated random-number table was used to generate the allocation sequence. Second, none of the studies mentioned a blinding method, but we interviewed the authors by telephone and found that 10 studies [15–17, 20–24, 26, 27] used single blinding (i.e., the outcome assessment was blinded), and four [18, 19, 25, 28] did not. The lack of blinding participants, health-care providers, and assessors can introduce performance and detection bias. Third, none of the studies addressed the incomplete outcome data, such as missing data due to attrition or exclusion. The inadequate handling of missing data can compromise statistical analyses. Fourth, the majority of experimental Chinese herbal medicine interventions were prepared by the investigators without detailed information describing their underlying rationale for the formulation, dosage, or the manufacturing process, and the quality control processes for their tested interventions are unknown. Thus, independent validation of these findings is necessary.

This paper has several methodological limitations. First, although all data were collected from reports or from direct contact with the authors, many items on the “risk of bias” assessment tool could only be classified as “unclear.” Second, different Chinese herbal medicine interventions were grouped together for analysis in some cases. The results might have been compromised by the heterogeneity within each Chinese herbal medicine intervention and the study design. Third, the concept of TCM syndrome was not considered when analyzing the data, as all studies only considered Childhood Pneumonia, not TCM symptoms. Therefore, the actual therapeutic effect might not have been fully captured. 

Based on these reasons, the TCM RCTs should be conducted in accordance with the Consolidated Standards of Reporting Trials for Traditional Chinese Medicine [29] detailed report.

## 7. Conclusions

Chinese herbal medicines may increase total effective rates, improve clinical symptoms and signs, and shorten the length of hospital stay for children with pneumonia. In a word, Chinese herbal medicines are effective for Childhood Pneumonia. However, there is insufficient evidence to confirm whether Chinese herbal medicines decrease adverse effects, mortality, and TCM outcomes (such as tongue coat and pulse condition). All results were supported by poor methodological quality studies. Thus, larger, multicenter, high methodological quality studies of Chinese herbal medicines for Childhood Pneumonia are needed. These studies should include patients with Childhood Pneumonia and interventions with Chinese herbal medicines. More data, particularly concerning adverse events, are necessary. Meanwhile, future studies should determine the most appropriate drug and dosage for Childhood Pneumonia. So, further trials would help to clarify the validity of the findings of this paper and could determine more clearly the role of Chinese herbal medicines in Childhood Pneumonia in comparison with other therapies. Consequently, the purpose is to guide our application in clinical.

## Figures and Tables

**Table 1:** Contents of the formulations used and the three languages are included in the included studies.

Study ID	Herbs (composition) in three languages	Method of administration
Wang et al., 2009 [15]	Modified Ma Xing Shi Gan Tang: Mahuang (Herba Ephedrae/Ephedra Herb), Xingren (Armeniacae Amarum/Bitter Apricot Seed), Shigao (Gypsum Fibrosum/Gypsum), Gancao (Radix Glycyrrhizae/LiquoriceRoot), Yuxingcao (Herba Houttuyniae/Heartleaf Houttuynia Herb), lianqiao (Fructus Forsythiae/WeepingForsythiaecapsule), Chanyi (Periostracum Cicadae/Cicada Slough), and Niupangzi (Fructus Arctii/Great Burdock Achene)	Oral administration

Zhao and Ji, 2009 [16]	Modified Ma Xing Shi Gan Tang: Mahuang (Herba Ephedrae/Ephedra Herb), Xingren (Armeniacae Amarum/Bitter Apricot Seed), Shigao (Gypsum Fibrosum/Gypsum), Gancao (Radix Glycyrrhizae/Liquorice Root), Yuxingcao (Herba Houttuyniae/Heartleaf Houttuynia Herb), and Huangqin (Radix Astragali Root)	Oral administration

Guo, 1999 [17]	Modified Ma Xing Shi Gan Tang: Mahuang (Herba Ephedrae/Ephedra Herb), Xingren (Armeniacae Amarum/Bitter Apricot Seed), Shigao (Gypsum Fibrosum/Gypsum), Gancao (Radix Glycyrrhizae/Liquorice Root), Yuxingcao (Herba Houttuyniae/Heartleaf Houttuynia Herb), and lianqiao (Fructus Forsythiae/WeepingForsythiaeCapsule)	Oral administration

Zhang, 2012 [18]	Modified Ma Xing Shi Gan Tang: Mahuang (Herba Ephedrae/Ephedra Herb), Xingren (Armeniacae Amarum/Bitter Apricot Seed), Shigao (Gypsum Fibrosum/Gypsum), Gancao (Radix Glycyrrhizae/Liquorice Root), Yuxingcao (Herba Houttuyniae/Heartleaf Houttuynia Herb), and Huangqin (Radix Astragali Root)	Oral administration

He, 2011 [19]	Modified Ma Xing Shi Gan Tang: Mahuang (Herba Ephedrae/Ephedra Herb), Xingren (Armeniacae Amarum/Bitter Apricot Seed), Jinhua (Flos Lonicerae/Honeysuckle Flower), Yinhua (Flos Lonicerae/Honeysuckle Flower), Shigao (Gypsum Fibrosum/Gypsum), Yuxingcao (Herba Houttuyniae/Heartleaf Houttuynia Herb), Banxia (Rhizome/Pinellia Tuberifera Tenora), and Gancao (Radix Glycyrrhizae/Liquorice Root)	Oral administration

He et al., 2011 [20]	Modified Ma Xing Shi Gan Tang: Mahuang (Herba Ephedrae/Ephedra Herb), Xingren (Armeniacae Amarum/Bitter Apricot Seed), Shigao (Gypsum Fibrosum/Gypsum), Suzi (Fructus Perillae/PerillaFruit), Shangbaipi (Cortex Mori/White Mulberry Root Bark), Kuandonghua (Flos Farfarae/Common Coltsfoot Flower), Banxia (Rhizome/Pinellia Tuberifera Tenora), Tinglizi (Semen Lepidii/SemenDescurainiae Pepperweed Seed/Tansymustard), Yuxingcao (Herba Houttuyniae/Heartleaf Houttuynia Herb), and Gancao (Radix Glycyrrhizae/Liquorice Root)	Oral administration

Zhang, 2012 [21]	San Ao Tang: Mahuang (Herba Ephedrae/Ephedra Herb), Xingren (Armeniacae Amarum/Bitter Apricot Seed), and Gancao (Radix Glycyrrhizae/Liquorice Root)	Oral administration

Wang et al., 2009 [22]	Modified Zhi Sou San: Jiegeng (Radix Platycodi/Platycodon Root), Gancao (Radix Glycyrrhizae/Liquorice Root), Ziwan (Radix Asteris/Tatarian Aster Root), Chenpi (Pericarpium Citri Reticulatae/Tangerine Peel), Xingren (Armeniacae Amarum/Bitter Apricot Seed), Baiguo (Semen Gingko/Ginkgo Seed), Huangqi (Radix Astragali Root), Chaomaiya (Fructus Hordei Germina/Malt), Yunling (Poria/Indian Buead), and Baiqian (Rhizoma Cynanchi Stauntonii/Willowleaf Swallowwort Rhizome/Glaucescent)	Oral administration

Lv et al., 2009 [23]	Self-Developed TCM Prescription: Mahuang (Herba Ephedrae/Ephedra Herb), Xingren (Armeniacae Amarum/Bitter Apricot Seed), Rengongniuhuang (Calculus Bovis/Bezoar), Baiqian (Rhizoma Cynanchi Stauntonii/Willowleaf Swallowwort Rhizome/Glaucescent), Shigao (Gypsum Fibrosum/Gypsum), Zhusha (Cinnabaris/Cinnabar), Chuanbeimu (Bulbus Fritillariae Unibracteatae/Unibract Fritillary Bulb), Huanglian (Rhizoma Coptidis/Golden Thread), Banxia (Rhizome/Pinellia Tuberifera Tenora), Dannanxing (Pinellia Pedatisecta/Arisaema with Bile), Shangbaipi (Cortex Mori/White Mulberry Root Bark), Huangqin (Radix Astragali Root), and Gancao (Radix Glycyrrhizae/Liquorice Root)	Oral administration

Lei, 2010 [24]	Tanreqing injection: no information provided about Tanreqing composition	Intravenous injection

Shi, 2009 [25]	Reduning injection: no information provided about Reduning composition	Intravenous injection

Pan, 2011 [26]	Reduning injection: no information provided about Reduning composition	Intravenous injection

Duan and Feng, 2011 [27]	Reduning injection: no information provided about Reduning composition	Intravenous injection

Wei and Feng, 2003 [28]	Chuanhuning injection: no information provided about Chuanhuning composition	Intravenous injection

**Table 2:** Characteristics of the included studies.

Wang et al., 2009 [15]	
	Randomized controlled trial (RCT): randomization mentioned, but not described in detail
	Allocation concealment: not mentioned
	Followup: not mentioned
Methods	Study duration: 3 weeks
	Parallel/crossover/factorial RCT: parallel
	Randomization method: we interviewed the author by telephone and learned that a random number table was used to generate the random sequence
	Blinding: no detailed information on blindness was offered. A telephone interview with the author revealed that single blinding was used
	ITT: not mentioned
	Setting: inpatients
Participants	Country: China
	Number: 106 patients with childhood pneumonia
	54 boys (50.8%) and 52 girls (49.2%); age 3–14 years old; disease duration: not mentioned
Interventions	Treatment group: modified Ma Xing Shi Gan Tang formula plus basic therapy: Mahuang 6 g, Xingren 8 g, Shigao 15 g, Gancao 5 g, Yuxingcao 20 g, lianqiao 15 g, Chanyi 10 g, and Niupangzi 15 g boiled in 3 L water and decocted to 300 mL. Orally twice daily (bid) for 3 weeks
	Control group: basic therapy including intravenous infusion of azithromycin and azithromycin orally
Outcomes	(1) Total effective rate
	(2) Adverse effects (e.g., nausea, diarrhea, and vomit)
Notes	(1) Duration of disease: not mentioned; (2) mortality: not mentioned; (3) relapse rate: not mentioned; (4) length of hospital stay: not mentioned; (5) clinical recovery (e.g., cough, fever, rales, and chest films): not mentioned; (6) TCM outcomes, such as tongue coat and pulse condition: not mentioned; (7) economic index: not mentioned; (8) withdrawal rates: not specified; (9) source of funding: none

Zhao and Ji, 2009 [16]	

	RCT: randomization mentioned, but not described in detail
	Allocation concealment: not mentioned
	Followup was not mentioned
Methods	Study duration: not mentioned
	Parallel/crossover/factorial RCT: parallel
	Randomization method: a telephone interview with the author revealed that a random number table was used to generate the random sequence
	Blinding: no detailed information on blindness was offered. A telephone interview with the author revealed that single blinding was used
	ITT: not mentioned
	Setting: inpatients and outpatients
	Country: China
Participants	Number: 60 patients with childhood pneumonia
	Treatment group: 30 patients with childhood pneumonia: 16 boys (53%) and 14 girls (47%); age: 2 months–9 years (mean: 3.5 years); disease duration: 4.00 ± 1.55 years
	Control group: 30 patients with childhood pneumonia: 17 boys (56.6%) and 13 girls (43.4%); age: month –11 years (mean: 3.25 years); disease duration: 4.00 ± 1.75 years
Interventions	Treatment group: modified Ma Xing Shi Gan Tang formula plus basic therapy: Mahuang 6 g, Xingren 10 g, Shigao 20 g, Gancao 3 g, Yuxingcao 10 g, and Huangqin 10 g boiled in 3 L water and decocted to 300 mL. Orally twice daily (bid) for 3 weeks
	Control group: basic therapy including intravenous infusion of azithromycin and azithromycin orally
Outcomes	(1) Total effective rate
	(2) Clinical recovery (e.g., cough, fever, and rales)
Notes	(1) Mortality: not mentioned; (2) relapse rate: not mentioned; (3) length of hospital stay: not mentioned; (4) TCM outcomes, such as tongue coat and pulse condition: not mentioned; (5) clinical recovery (e.g., chest films): not mentioned; (6) economic index: not mentioned; (7) withdrawal rates: not mentioned; (8) source of funding: none;(9)adverse effects (e.g., nausea, diarrhea, and vomiting): not mentioned

Guo, 1999 [17]	

	RCT: randomization mentioned, but not described in detail
	Allocation concealment: not mentioned
	Followup: not mentioned
Methods	Not mentioned
	Parallel/crossover/factorial RCT: parallel
	Randomization method: a telephone interview with the author revealed that a random number table was used to generate the random sequence
	Blinding: no detailed information on blindness was offered. A telephone interview with the author revealed that single-blinding was used
	ITT: not mentioned
	Setting: not mentioned
	Country: China
Participants	Number: 170 patients with childhood pneumonia
	Treatment group: 86 patients with childhood pneumonia: 45 boys (52.3%) and 41 girls (48.7%); age: 2 months–12 years (mean: 3.8 years)
	Control group: 84 patients with childhood pneumonia: 44 boys (52.4%) and 40 girls (48.6%); age: 2 months–14 years (mean: 3.2 years)
Interventions	Treatment group: modified Ma Xing Shi Gan Tang formula plus basic therapy. Mahuang 1.5 g, Xingren 3 g, Shigao 10 g, Gancao 1.5 g, Yuxingcao 9 g, and lianqiao 3 g, boiled in 3 L water and decocted to 300 mL. Taken orally, three times daily (tid)
	Control group: basic therapy included penicillin, Xianfeng Meisu, and ribavirin. Intravenous infusion of azithromycin and azithromycin orally
Outcomes	(1) Total effective rate
Notes	(1) Mortality: not mentioned; (2) relapse rate: not mentioned; (3) length of hospital stay: not mentioned; (4) TCM outcomes, such as tongue coat and pulse condition: not mentioned; (5) economic index: not mentioned; (6) withdrawal rates: not specified; (7) source of funding: None; (8) time to measure outcomes: not mentioned; (9) 2. clinical recovery (e.g., cough, fever, rales, and chest films): not mentioned

Zhang, 2012 [18]	

	RCT: randomization mentioned, but not described in detail
	Allocation concealment: not mentioned
	Followup: not mentioned
Methods	Study duration: 20 days
	Parallel/crossover/factorial RCT: parallel
	Randomization method: a telephone interview with the author revealed that a random number table was used to generate the random sequence
	Blinding: no detailed information on blindness was offered. Telephone interview with author revealed that blinding was not used
	ITT: not mentioned
	Setting: not mentioned
	Country: China
Participants	Number: 200 patients with childhood pneumonia
	Treatment group: 100 patients with childhood pneumonia: 74 boys (74%) and 26 girls (47%); mean age: 6.28 years; disease duration: not mentioned
	Control group: 100 patients with childhood pneumonia: 68 boys (68%) and 32 girls (32%); age and duration of disease not mentioned
Interventions	Treatment group: modified Ma Xing Shi Gan Tang formula plus basic therapy. Mahuang 3 g, Xingren 4 g, Shigao 18 g, Gancao 3 g, Yuxingcao 9 g, and Huangqin 3 g boiled in 3 L water and decocted to 300 mL, taken orally twice daily (bid)
	Control group: basic therapy included intravenous infusion of azithromycin and azithromycin orally
Outcomes	(1) Total effective rate
	(2) Clinical recovery (e.g., cough, fever, rales, and chest films)
Notes	(1) Mortality: not mentioned; (2) relapse rate: not mentioned; (3) length of hospital stay: not mentioned; (4) TCM outcomes, such as the tongue coat and pulse condition: not mentioned; (5) economic index: not mentioned; (6) withdrawal rates: not mentioned; (7) source of funding: none; (8) time to measure outcomes: not mentioned; (9) adverse effects: not mentioned

He, 2011 [19]	

	RCT: randomization mentioned, but not described in detail
	Allocation concealment: not mentioned
	Followup: not mentioned
Methods	Study duration: 5–7 days
	Parallel/crossover/factorial RCT: parallel
	Randomization method: a telephone interview with the author revealed that a random number table was used to generate the random sequence
	Blinding: no detailed information on blindness was offered. A telephone interview with the author revealed that blinding was not used
	ITT: not mentioned
	Setting: inpatients
	Country: China
Participants	Number: 100 patients with childhood pneumonia
	Treatment group: 50 patients with childhood pneumonia: 29 boys (54%) and 21 girls (42%); age: 9.6 months–12 years; disease duration: 4–8.5 days
	Control group: 50 patients with childhood pneumonia: 27 boys (54%) and 23 girls (46%); age: 10.8 months–13 years; disease duration: 5–8 days
Interventions	Treatment group: modified Ma Xing Shi Gan Tang formula plus basic therapy: Mahuang 3 g, Xingren 6 g, Jinhua 6 g, Yinhua 6 g, Shigao 12 g, Yuxingcao 9 g, Banxia 6 g, and Zhigancao 3 g boiled in 2 L water and decocted to 300 mL; taken orally twice daily (bid)
	Control group: intravenous infusion of azithromycin (10 mg/k · d)
Outcomes	(1) Total effective rate
Notes	(1) Mortality: not mentioned; (2) relapse rate: not mentioned; (3) length of hospital stay: not mentioned; (4) TCM outcomes, such as tongue coat and pulse condition: not mentioned; (5) economic index: not mentioned; (6) withdrawal rates: not specified; (7) source of funding: none; (8) time to measure outcomes: not mentioned; (9) adverse effects: not mentioned; (10) clinical recovery (e.g., cough, fever, rales, and chest films): not mentioned

He et al., 2011[20]	

	RCT: randomization mentioned, but not described in detail
	Allocation concealment: not mentioned
	Followup: not mentioned
Methods	Study duration: 7 days
	Parallel/crossover/factorial RCT: parallel
	Randomization method: a telephone interview with the author revealed that a random number table was used to generate the random sequence
	Blinding: no detailed information on blindness was offered. A telephone interview with the author revealed that blinding was used on outcome assessment
	ITT: not mentioned
	Setting: inpatients
	Country: China
Participants	Number: 80 patients with childhood pneumonia
	Treatment group: 40 patients with childhood pneumonia: 22 boys (55%) and 18 girls (45%); mean age: 1.8 ± 1.10 years
	Control group: 40 patients with childhood pneumonia: 21 boys (52.5%) and 19 girls (47.5%); mean age: 1.75 ± 1.151 years
	Disease duration: 7.50 ± 0.50 days
Interventions	Treatment group: modified Ma Xing Shi Gan Tang formula plus basic therapy: Mahuang 3 g, Xingren 3 g, Shigao 9 g, Suzi 3 g, Shangbaipi 6 g, Kuandonghua 6 g, Banxia 6 g, Tinglizi 3 g, Yuxingcao 3 g, and Gancao 3 g boiled in 2 L water and decocted to 300 mL, taken orally twice daily (bid)
	Control group: basic therapy included supporting treatment and intravenous infusion of ceftazidime (0.1 g/kg · d)
Outcomes	(1) Total effective rate
Notes	(1) Mortality: not mentioned; (2) relapse rate: not mentioned; (3) length of hospital stay: not mentioned; (4) TCM outcomes, such as tongue coat and pulse condition: not mentioned; (5) adverse effects: not mentioned; (6) economic index: not mentioned; (7) withdrawal rates: not mentioned; (8) source of funding: none; (9) time to measure outcomes: not mentioned; (10) clinical recovery (e.g., cough, fever, rales, and chest films): not mentioned

Zhang, 2012 [21]	

	RCT: randomization mentioned, but not described in detail
	Allocation concealment: not mentioned
	Followup: not mentioned
Methods	Study duration: 5–7 days
	Parallel/crossover/factorial RCT: parallel
	Randomization method: a telephone interview with the author revealed that a computer-generated random-number table was used
	Blinding: no detailed information on blindness was offered. A telephone interview with the author revealed that blinding was used on the outcome assessment
	ITT: not mentioned
	Setting: inpatients
	Country: China
Participants	Number: 128 patients with childhood pneumonia
	Treatment group: 64 patients with childhood pneumonia in the treatment group
	Control group: 64 patients with childhood pneumonia in the control group
	In two groups, 79 boys (55%) and 49 girls (45%); age 1–14 years old; disease duration: 5–7 days
Interventions	Treatment group: San Ao Tang formula plus basic therapy; Mahuang 3 g, Xingren 12 g, Gancao 3 g boiled in 2 L water and decocted to 250 mL. Taken orally three times daily (tid)
	Control group: basic therapy included symptomatic therapy and orally azithromycin 10 mg/kg one time per day (qd)
Outcomes	(1) Total effective rate; (2) clinical recovery (e.g., cough, fever, rales, and chest films); (3) adverse effects (e.g., nausea)
Notes	(1) Mortality: not mentioned; (2) relapse rate: not mentioned; (3) length of hospital stay: not mentioned; (4) TCM outcomes, such as tongue coat and pulse condition: not mentioned; (5) economic index: not mentioned; (6) withdrawal rates: not mentioned; (7) source of funding: none; (8) time to measure outcomes: not mentioned

Wang et al., 2009 [22]	

	RCT: randomization mentioned, but not described in detail
	Allocation concealment: not mentioned
	Followup: not mentioned
	Study duration: 7 days
Methods	Parallel/crossover/factorial RCT: parallel
	Randomization method: a telephone interview with the author revealed that a computer-generated random-number table was used
	Blinding: no detailed information on blindness was offered. A telephone interview with the author revealed that blinding was used on outcome assessment
	ITT: not mentioned
	Setting: patient source not mentioned
	Country: China
	Number: 200 patients with childhood pneumonia
Participants	Treatment group: 100 patients with childhood pneumonia in the treatment group
	Control group: 100 patients with childhood pneumonia in the control group
	Did not mention the number of boys and girls
	Disease duration: 5–7 days
Interventions	Treatment group: Zhi Sou San formula plus basic therapy: Jiegeng 6–9 g, Gancao 3–6 g, Ziwan 3–6 g, Chenpi 3–6 g, Xingren 3–6 g, Baiguo 3–6 g, Huangqi 3–6 g, Chaomaiya 3–6 g, Yunling 3–6 g, and Baiqian 3–6 g boiled in 2 L water. Taken orally three times daily (tid)
	Control group: basic therapy included symptomatic therapy and intravenous infusion of erythrocin (30 mg/kg · d)
Outcomes	(1) Total effective rate; (2) clinical recovery (e.g., cough, fever, and rales); (3) adverse effects (e.g., nausea, vomiting, and gastrointestinal bleeding)
Notes	(1) Mortality: not mentioned; (2) relapse rate: not mentioned; (3) length of hospital stay: not mentioned; (4) TCM outcomes, such as tongue coat and pulse condition: not mentioned; (5) economic index: not mentioned; (6) withdrawal rates: not mentioned; (7) source of funding: none; (8) time to measure outcomes: not mentioned; (9) chest films: not mentioned

Lv et al., 2009 [23]	

	RCT: randomization mentioned, but not described in detail
	Allocation concealment: not mentioned
	Followup was not mentioned
Methods	Study duration: 10 days
	Parallel/crossover/factorial RCT: parallel
	Randomization method: a telephone interview with the author revealed that a random number table was used to generate the random sequence
	Blinding: no detailed information on blindness was offered. A telephone interview with the author revealed that blinding was used on the outcome assessment
	ITT: not mentioned
	Setting: inpatients
	Country: China
Participants	Number: 60 patients with childhood pneumonia
	Treatment group: 30 patients with childhood pneumonia: 17 boys (56.7%) and 13 girls (43.3%); age: 8 months–13 years; disease duration: 7–18 days; mean: 14 days
	Control group: 30 patients with childhood pneumonia: 16 boys (53.3%) and 14 girls (46.7%); age: 7 months–12 years; disease duration: 7–18 days; mean: 13.5 days
Interventions	Treatment group: self-developed TCM prescription plus basic therapy: Mahuang 3 g, Xingren 3 g, Rengongniuhuang 3 g, Bingpian 2 g, Shengshigao 3 g, Zhusha 2 g, Chuanbeimu 2 g, Huanglian 2 g, Banxia 2 g, Dannanxing 2 g, Shangbaipi 2 g, Huangqin 2 g, and Gancao 2 g boiled in 3 L water and decocted to 300 mL. Taken orally three times daily (tid)
	Control group: basic therapy included symptomatic therapy and intravenous infusion of erythrocin (20 mg/kg bid)
Outcomes	(1) Total effective rate; (2) clinical recovery (e.g., cough, fever, rales, and chest films)
Notes	(1) Mortality: not mentioned; (2) relapse rate: not mentioned; (3) length of hospital stay: not mentioned; (4) TCM outcomes, such as tongue coat and pulse condition: not mentioned; (5) economic index: not mentioned; (6) withdrawal rates: not mentioned; (7) source of funding: none; (8) time to measure outcomes: mentioned; (9) adverse effects: not mentioned

Lei, 2010 [24]	

	RCT: randomization mentioned, but not described in detail
	Allocation concealment: not mentioned
	Followup: not mentioned
Methods	Study duration: 7 days
	Parallel/crossover/factorial RCT: parallel
	Randomization method: a telephone interview with the author revealed that a computer-generated random-number table was used
	Blinding: no detailed information on blindness was offered. A telephone interview with the author revealed that blinding was used on the outcome assessment
	ITT: not mentioned
	Setting: inpatients
	Country: China
Participants	Number: 160 patients with childhood pneumonia
	Treatment group: 80 patients with childhood pneumonia: 52 boys (65%) and 28 girls (35%); age: 6 months–12 years; disease duration: 2–7 days
	Control group: 80 patients with childhood pneumonia: 54 boys (67.5%) and 26 girls (32.5%); age: 5 months–13 years; disease duration: 1–6 days
Interventions	Treatment group: Tanreqing injection plus basic therapy: 30–50 mL/kg Tanreqing injection + 50–100 mL 10% GS intravenous infusion once daily (qd)
	Control group: basic therapy included anti-inflammatory, symptomatic therapy. Did not provide any detailed information about the anti-inflammatory, symptomatic therapy
Outcomes	(1) Total effective rate; (2) clinical recovery (e.g., cough, fever, and rales).
Notes	(1) Mortality: not mentioned; (2) relapse rate: not mentioned; (3) length of hospital stay: not mentioned; (4) TCM outcomes, such as tongue coat and pulse condition: not mentioned; (5) economic index: not mentioned; (6) withdrawal rates: not mentioned; (7) source of funding: none; (8) time to measure outcomes: mentioned; (9) chest films: not mentioned; (10) adverse effects: not mentioned

Shi, 2009 [25]	

	RCT: randomization mentioned, but not described in detail
	Allocation concealment: not mentioned
	Followup: not mentioned
Methods	Study duration: 14 days
	Parallel/crossover/factorial RCT: parallel
	Randomization method: a random number table was used to generate the random sequence
	Blinding: no detailed information on blindness was offered. A telephone interview with the author revealed that blinding was not used on study
	ITT: not mentioned
	Setting: inpatients
	Country: China
Participants	Number: 80 patients with childhood pneumonia
	Treatment group: 40 patients with childhood pneumonia: 21 boys (52.5%) and 19 girls (47.5%)
	Control group: 40 patients with childhood pneumonia: 24 boys (60%) and 16 girls (40%); age: 5 months–13 years, in two groups; disease duration: 1–3 days
Interventions	Treatment group: Reduning injection plus basic therapy: 0.5–1.0 mL/kg · d Reduning injection + 250 mL 5% GS intravenous infusion once daily (qd)
	Control group: basic therapy included symptomatic therapy and intravenous infusion of 10 mg/kg · d azithromycin for 5 days, stop 3 days, then changed to oral 10 mg/kg · d azithromycin for 3 days
Outcomes	(1) Total effective rate; (2) clinical recovery (e.g., cough, fever, rales, and chest films)
Notes	(1) Mortality: not mentioned; (2) relapse rate: not mentioned; (3) length of hospital stay: not mentioned; (4) TCM outcomes, such as tongue coat and pulse condition: not mentioned; (5) economic index: not mentioned; (6) withdrawal rates: not mentioned; (7) source of funding: none; (8) time to measure outcomes: mentioned; (9) adverse effects: not mentioned

Pan, 2011 [26]	

	RCT: randomization mentioned, but not described in detail
	Allocation concealment: not mentioned
	Followup: not mentioned
Methods	Study duration: 7 days
	Parallel/crossover/factorial RCT: parallel
	Randomization method: a telephone interview with the author revealed that a random number table was used to generate the random sequence
	Blinding: no detailed information on blindness was offered. A telephone interview with the author revealed that the outcome assessment was blinding
	ITT: not mentioned.
	Setting: patient source not mentioned.
	Country: China
Participants	Number: 140 patients with childhood pneumonia
	Treatment group: 70 patients with childhood pneumonia: 42 boys (60%) and 28 girls (40%); age: 1–9 years old (mean: 5.1 ± 1.6 years); disease duration: 2–7 days
	Control group: 70 patients with childhood pneumonia: 36 boys (51.4%) and 34 girls (48.6%); age: 1–10 years old (mean: 5.1 ± 1.6 years); disease duration: 1–7 days
Interventions	Treatment group: Reduning injection plus basic therapy: 0.5–0.8 mL/kg Reduning injection + 100 mL 5% GS intravenous infusion once daily (qd)
	Control group: intravenous infusion of 10 mg/kg · d azithromycin + 5% GS once daily (qd)
Outcomes	(1) Total effective rate; (2) clinical recovery (e.g., cough, fever, and rales)
Notes	(1) Mortality: not mentioned; (2) relapse rate: not mentioned; (3) length of hospital stay: not mentioned; (4) TCM outcomes, such as tongue coat and pulse condition: not mentioned; (5) economic index: not mentioned; (6) withdrawal rates: not mentioned; (7) source of funding: mentioned; (8) time to measure outcomes: not mentioned; (9) adverse effects: not mentioned; (10) chest films: not mentioned

Duan and Feng, 2011 [27]	

	RCT: randomization mentioned, but not described in detail
	Allocation concealment: not mentioned
	Followup was not mentioned
Methods	Study duration: 14 days
	Parallel/crossover/factorial RCT: parallel
	Randomization method: a telephone interview with the author revealed that a random number table was used to generate the random sequence
	Blinding: no detailed information on blindness was offered. A telephone interview with the author revealed that the outcome assessment was blinded
	ITT: not mentioned
	Setting: outpatients and inpatients
Participants	Country: China
	Number: 60 patients with childhood pneumonia
	In two groups: 35 boys (58.3%) and 25 girls (41.7%); age: 1–13 years; disease duration: 2–5 days
Interventions	Treatment group: Reduning injection plus basic therapy: 10–15 mL Reduning injection + 100 mL 5% GS intravenous infusion once daily (qd)
	Control group: intravenous infusion of 10 mg/kg · d azithromycin + 5% GS once daily (qd)
Outcomes	(1) Total effective rate; (2) clinical recovery (e.g., fever)
Notes	(1) Mortality: not mentioned; (2) relapse rate: not mentioned; (3) length of hospital stay: not mentioned; (4) TCM outcomes, such as tongue coat and pulse condition: not mentioned; (5) economic index: not mentioned; (6) clinical recovery (e.g., cough, rales, and chest films): not mentioned; (7) withdrawal rates: not mentioned; (8) source of funding: none; (9) time to measure outcomes: not mentioned; (10) adverse effects: not mentioned

Wei and Feng, 2003 [28]	

	RCT: randomization mentioned, but not described in detail
	Allocation concealment: not mentioned
	Followup: not mentioned
Methods	Study duration: 7–10 days
	Parallel/crossover/factorial RCT: parallel
	Randomization method: a telephone interview with the author revealed that a computer-generated random-number table was used
	Blinding: no detailed information on blindness was offered. A telephone interview with the author revealed that blinding was not used
	ITT: not mentioned
	Setting: inpatients
	Country: China
Participants	Number: 180 patients with childhood
	In two groups: 100 boys (55.6%) and 80 girls (44.4%); age: 2 months–5 years (mean: 2.3 years); disease duration: 1–7 days
	Treatment group: 90 patients with childhood pneumonia; 52 boys (57.8%) and 38 girls (42.2%)
	Control group: 90 patients with childhood pneumonia; 48 boys (53.3%) and 42 girls (46.7%)
Interventions	Treatment group: Chuanhuning injection plus basic therapy: 10 mg/kg · d + 50–100 mL 10% GS or NS intravenous infusion once daily (qd)
	Control group: intravenous infusion of 100 mg/kg · d piperacillin twice daily (bid)
Outcomes	(1) Total effective rate; (2) length of hospital stay; (3) clinical recovery (e.g., cough, fever, rales, and chest films)
Notes	(1) Mortality: not mentioned; (2) relapse rate: not mentioned; (3) TCM outcomes, such as tongue coat and pulse condition: not mentioned; (4) economic index: not mentioned; (5) withdrawal rates: not mentioned; (6) source of funding: none; (7) time to measure outcomes: not mentioned; (8) adverse effects: not mentioned

**Table 3:** Grade quality of evidence.

Chinese medicinal herbs plus basic therapy versus basic therapy alone for childhood pneumonia						
Patient or population: patients with childhood pneumonia						
Settings: inpatients or outpatients						
Intervention: Chinese medicinal herbs plus basic therapy versus basic therapy alone						
	Illustrative comparative risks (95% CI)*					
Outcomes	Assumed risk	Corresponding risk	Relative effect (95% CI)	Number of Participants (studies)	Quality of evidence (grade)	Comments
	Control	Chinese medicinal herbs plus basic therapy versus basic therapy alone				

	Study population					
Total effective rate	**779 per 1000**	**920 per 1000** (865–982)				
	Moderate		**RR 1.18** (1.11–1.26)	1720 (14 studies)	⊕⊕○○ **Low** ^1,2^	Important
	**800 per 1000**	**944 per 1000** (888–1000)				

	Study population					
Adverse effects	**419 per 1000**	**164 per 1000** (38–721)				
	Moderate		**RR 0.39** (0.09–1.72)	434 (3 studies)	⊕○○○ **Very low** ^1,3^	Important
	**113 per 1000**	**44 per 1000** (10 to 194)				

Time (day) to improvement of cough		The mean time (days) to improvement in cough in the intervention groups was **2.18 lower** (2.66 to 1.71 lower)		1208 (9 studies)	⊕⊕○○ **Low** ^1,4^	Important

Time (day) to improvement of fever		The mean time (days) to improvement in fever in the intervention groups was **2.12 lower** (2.25 to 1.98 lower)		1262 (10 studies)	⊕⊕○○ **Low** ^1,2^	Important

Time (day) to improvement of rales		The mean time (days) to improvement in rales in the intervention groups was **1.53 lower** (1.84 to 1.23 lower)		1208 (9 studies)	⊕⊕○○ **Low** ^1,4^	Important

Time (day) to improvement in chest films		The mean time (days) to improvement in chest films in the intervention groups was **3.1 lower** (4.11 to 2.08 lower)		648 (5 studies)	⊕⊕○○ **Low** ^1,4^	Important

Length of hospital stay		The mean length of hospital stay in the intervention groups was **3 lower** (3.52 to 2.48 lower)		180 (1 study)	⊕○○○ **Very low** ^1,5^	Important

*The basis for the assumed risk (e.g., the median control group risk across studies) is provided in the footnotes. The corresponding risk (and its 95% confidence interval) is based on the assumed risk in the comparison group and the relative effect of the intervention (and its 95% CI). CI: confidence interval; RR: risk ratio.

Grade: working group grades of evidence.

**High quality:** further research is very unlikely to change our confidence in the estimate of effect. Handbook description: randomized controlled trial.

**Moderate quality:** further research is likely to have an important impact on our confidence in the estimate of effect and may change the estimate. Cochrane Handbook description: relegation randomized controlled trial.

**Low quality:** further research is very likely to have an important impact on our confidence in the estimate of effect and is likely to change the estimate. Cochrane Handbook description: two or more degradation factors of randomized controlled trials.

**Very low quality:** we are very uncertain about the estimate. Cochrane Handbook description: more than three degradation factors of randomized controlled trials.

Reduce the evidence quality factors: methodology defect, included in the research results of the inconsistency, indirect evidence, inexactness, and publication bias.

Increase the level of evidence factor: large effect quantity, confounding factors cannot change effect quantity, or the existing concentration-response relationship.

^1^There is a high risk of selection bias, performance bias, and detection bias.

^2^Some studies showed a significant difference, but some studies showed no significant difference.

^3^Few studies included.

^4^The protocol of the published studies could not be compared.

^5^Only one study included.
